# Role of maternal sound stimulation in the NICU: improving outcomes for newborns and their families

**DOI:** 10.3389/fped.2026.1764698

**Published:** 2026-03-20

**Authors:** Monali Runa Mukerji, Sunitha Vimalesvaran, Christelle Sanchez, Minesh Khashu

**Affiliations:** 1 Department of Pediatrics, National University Hospital, Singapore, Singapore; 2 Incubeats Tech Ltd, London, United Kingdom; 3 Neonatal Unit, University Hospitals Dorset, Dorset, United Kingdom

**Keywords:** family integrated care, maternal, neonate, neurodevelopment, NICU, noise, preterm, sound stimulation

## Abstract

The Neonatal Intensive Care Unit (NICU) presents significant sensory and emotional challenges for preterm infants and their families. Early maternal separation, aberrant sensory exposures, and potential limitations to parental bonding pose risks to infants' neurodevelopment and overall health, whilst families may experience emotional distress. This study of current scientific literature evaluates the necessity and potential impact of using the recording of maternal sounds to simulate intrauterine auditory environments in NICUs. A narrative review of studies assessing sensory interventions in NICUs was conducted, focusing on outcomes related to infant neurodevelopment, family bonding, and caregiver experiences. Data were synthesized to determine the effectiveness and uniqueness of audio-stimulation of infants using recordings of maternal sounds compared to existing interventions. Maternal sound stimulation, including voice and heartbeat playback, has been shown to promote neurodevelopment by enhancing auditory cortex maturation, reducing stress responses, and improving breastfeeding outcomes in preterm infants. It may also help in reducing the incidence of cardiorespiratory events, mitigation of pain responses during procedures, and improvement in sleep regulation. While the benefits of maternal sound recordings are well established in specific areas, further research is needed to evaluate their long-term impact on cognitive development, emotional well-being, and attachment outcomes in preterm infants. Additionally, the broader effects of this intervention on family bonding and wellbeing, particularly in diverse NICU settings, require more extensive exploration. Recordings of maternal sounds hold significant promise as a targeted, practical solution to address the sensory needs of preterm infants and also help in strengthening the infant-family bond.

## 1 Introduction

Each year, approximately 8%–10% of all live births require admission to the Neonatal Intensive Care Unit (NICU) for varying durations, depending on the severity of the infant's condition or level of prematurity (1). While NICUs significantly enhance survival rates, current NICU practice may lead to or contribute to complications and long-term developmental consequences for babies e.g., through environmental noise, maternal separation, exposure to painful procedures, and disruptions in sleep patterns (2). The aim of this narrative review is to evaluate the current scientific literature regarding the potential impact of using the recordings of maternal sounds including heartbeats to simulate intrauterine auditory environments in NICUs.

## 2 Search strategy

The literature search covered publications from 1st January 2000 to 31st October 2024. We included peer-reviewed studies, published in the English language, evaluating maternal or parental sound stimulation (live or recorded), studies assessing NICU acoustic environments, and trials investigating physiological or neurodevelopmental effects of auditory interventions in the NICU. Exclusion criteria included studies unrelated to NICU settings, animal studies, and commentaries without original data. Relevant studies were identified after a comprehensive search from electronic databases (MEDLINE (Ovid), EMBASE (Elsevier), Cochrane Central Register of Controlled Trials (CENTRAL) (Wiley), Educational Resource Information Centre (ERIC) (Ovid)) and grey literature sources (Google Scholar). Studies were screened in parallel and relevant data was extracted independently by two review authors (SV, MRM). Any discrepancies were resolved through consensus or discussion with a third review author (MK).

As this was a narrative review, studies were selected for their conceptual and clinical relevance rather than through a formal systematic review process with a predefined quantitative inclusion threshold. 40 original studies specifically evaluating maternal or parental sound stimulation in the NICU informed the final narrative synthesis, alongside key background and developmental care literature to provide clinical and theoretical context. This approach allowed a comprehensive thematic overview while acknowledging the inherent risk of selection bias.

## 3 Early life exposure to the NICU

### 3.1 The impact of the NICU environment on neonates

One of the most concerning aspects of NICU care is the overwhelming exposure of neonates to unnatural sounds. The intrauterine environment provides a highly controlled acoustic landscape, where low-frequency maternal heartbeats, vascular flow, and muffled external noises create an optimal auditory setting for fetal development (3). Sounds experienced in the intrauterine environment are on an average ∼30 decibels (dB) quieter than in the extrauterine environment (3). Because high frequency (HF) and loud sounds are damaging to a developing infant, the absence of them in the womb is a reflection of the womb's protective nature. The intrauterine lining acts as a low-pass filter that absorbs HF sounds and attenuates all sounds (3). The protective nature of the womb is especially important in the third trimester. This is a period of rapid brain development, where the levels of growth are unparalleled at any other point in life (2). Indeed, two-thirds of brain growth occurs after 28 weeks gestational age (GA).

However, the NICU is markedly different, with high-intensity alarms, machine-generated sounds, and inconsistent human speech patterns. The American Academy of Pediatrics guidelines have recommended that sound does not exceed 45 dB (4). However, these recommendations do not protect the infant for two reasons. Firstly, the acoustic guidelines deal only with the issues of volume (loudness in dB), and not pitch [frequency in Hertz (Hz)] (5). Secondly, the guidelines are often ignored (6) as structural reformation of the NICU—for which there are insufficient funds—is often required for compliance (7). Some advocate that housing NICU infants in private, opposed to shared, rooms will solve the problem of aberrant sensory exposure in the NICU. However, sensory deprivation can be just as harmful as sensory overstimulation, and the problem is largely in the quality, not the quantity of the sensory exposure (8).

Studies have shown that high-frequency sounds can be stressful for neonates and may interfere with their auditory processing development (9). The lack of low-frequency maternal sounds in the NICU is believed to exacerbate sensory processing difficulties, leading to potential neurodevelopmental difficulties (10, 11).

Additionally pain is the unpleasant sensory and emotional experience associated with actual or potential tissue damage. It was originally supposed that infants’ underdeveloped nociceptive (pain-related) systems prevented them from feeling pain (12), and so anaesthetics were seldom used in this population. A paradigm shift occurred in the 1980s, and the knowledge that infants can feel pain from the 20th-24th week GA was better acknowledged (13, 14). In fact, we now know that the dysmaturity of the nociceptive system may render infants hypersensitive to pain (14), where even minor events such as being held firmly may be painful.

Another significant stressor for neonates in the NICU is maternal separation. Attachment theory highlights the importance of early maternal contact for establishing emotional security and cognitive development (15). Research indicates that neonates separated from their mothers exhibit elevated cortisol levels, indicating heightened stress and reduced physiological stability (16). Moreover, the absence of maternal voice exposure in early development has been linked to delayed language acquisition, impaired emotional regulation, and weakened social interaction skills later in life (17).

Ultimately, infants in the NICU face significant health risks, including high mortality rates that depend on gestational age, with survival rates ranging from 17% to 47% at 22 weeks to 92%–93% at 27 weeks (18). Those who survive have cardiorespiratory instability with apneas and bradycardias in the short term and neurodevelopment concerns in the long term. Hearing impairments are common due to early exposure to high-frequency sounds in the NICU (9, 19). Many also suffer from maternal breast milk deprivation, which can impact on the quality of their nutrition and immune protection (20, 21). Developmental delays, learning disabilities, and post-traumatic stress disorder are also connected with the NICU experience (22). Maternal separation disrupts attachment formation, leading to social and behavioural difficulties later in life (23). These cumulative challenges highlight the long-term impact of the NICU experience on infant health and development.

### 3.2 Implications of the NICU to the mothers and families of infants

Mothers of infants in the NICU often endure a traumatic birth experience, followed by distressing separation from their child, loss of maternal role, and public scrutiny. The inability to be with their baby due to physical recovery, hospital policies, or fragile neonatal conditions can be emotionally devastating (24). Many struggle with breastfeeding deprivation, which affects both their physical health and sense of maternal identity, as breastfeeding provides both medical benefits and emotional empowerment (25). The psychological toll includes high rates of post-traumatic stress disorder (PTSD), depression, and obsessive compulsive disorder (OCD), often rooted in feelings of helplessness and anxiety about their baby's well-being (26). Confidence loss is common, as mothers feel unprepared for caregiving after extended NICU stays, exacerbated by reliance on medical and nursing staff (27). Additionally, the strain on family relationships, particularly with spouses and other children, adds to the burden, sometimes increasing the risk of divorce and isolation (28). These compounded difficulties make the NICU experience profoundly challenging for mothers.

The impact on the family also cannot be underestimated. Fathers of NICU infants experience unique emotional and logistical challenges, balancing concern for both their child and partner while often being overlooked for support (28). The NICU environment itself can be distressing, and fathers frequently endure prolonged separation from their child due to cultural norms and work obligations. The burden of maintaining pre-birth responsibilities—such as employment and caring for siblings—adds to their stress, often forcing difficult choices regarding paternity leave (29). These pressures contribute to high rates of PTSD, with many fathers experiencing persistent mental health struggles post-NICU (26). A profound sense of helplessness, exacerbated by societal expectations that position mothers as primary caregivers, can lead to depression. Additionally, familial strain is common, as fathers juggle household responsibilities while feeling unsupported by partners focused on their own recovery. Similarly, siblings undergo significant disruption, as their routines shift, parental attention diminishes, and they struggle to process the NICU experience, sometimes manifesting confusion through play or emotional withdrawal (30).

## 4 Where do we stand?

To address these concerns, existing research is constantly exploring ways to improve the NICU experience for all parties. The needs of the infant center around the need for ‘neuroprotective developmental care’ (31) which includes the need to minimize pain, safeguard sleep, protect skin, optimize nutrition, and position and handle the infant carefully. Together with a good partnership with parents and the wider family, these aspects of care will lead to a healing environment for the infant, allowing them to prosper outside of the NICU. National guidelines are created off the back of these recommendations—such as caps on sensory stimuli in the NICUs and the encouragement of family-integrated care. Moreover, organizations, such as UNICEF, professional and various parental advocacy groups are creating optimization toolkits, nationally and globally, that are in various stages of implementation.

## 5 Maternal sound stimulation

### 5.1 Different modalities of maternal sound stimulation

Maternal sound stimulation is the use of a mother's live vocal interaction (e.g., talking, singing, or reading during skin-to-skin care or bedside presence), mother's voice recording, or sound of their heartbeat to provide auditory stimulation for preterm infants in the NICU. Mothers can record themselves singing, speaking or telling stories to their babies in NICU and these recordings can be played for the infant in their incubator. Research shows that this form of sound stimulation helps to promote physiological stability in the form of reduced apneas and bradycardias, as well as less fluctuation of oxygen saturation levels (32). In addition, it is considered to be a way of promoting mother-infant bonding as well as providing the infant with a sense of security and comfort whilst undergoing painful procedures in NICU. The sound of the mother's voice or heartbeat emulates what the infant would have been exposed to whilst inside the womb.

White noise is another well-known modality that can be used to comfort babies in the neonatal intensive care setting by mimicking the comforting sounds of the mother's womb. It is characterised by a mix of all audible frequencies to the human ear delivered at the same intensity. It is thought to promote relaxation and create a calming and familiar environment for the neonate by replicating the mixture of sound they would be exposed to *in utero*—e.g., digestive sounds, maternal heartbeat, the sound of blood flow all blended together (33).

In particular, researchers have explored the potential benefits of controlled maternal sound exposure in NICUs. Interventions such as playing recorded maternal heartbeats (Mimo Pillow) or voice messages (Mylittleone, Vcreate) or both (Incubeats) have shown promising results in reducing neonatal stress and promoting neurodevelopment (34–37).

It is beyond the scope of this review to explore in detail the pros and cons of these various modalities of nurturing sound stimulation for infants in the NICU. Moreover, the similar use of such technologies for using voice of the father, siblings and grandparents also requires consideration as it promotes family bonding. We will focus on the evidence regarding the benefits of maternal/parental sound stimulation in general.

### 5.2 Benefits of maternal sound stimulation

Physiological benefits include a reduction in cardiorespiratory events, with studies showing that maternal voice exposure decreases the frequency of apnea and bradycardia episodes in preterm infants (35). Additionally, preterm infants exposed to maternal heartbeat sounds exhibit more stable oxygen levels compared to those in conventional NICU settings (10). Research indicates that controlled exposure to maternal sounds contributes to a more regulated autonomic response, reducing erratic fluctuations in heart rate and respiration that are common in preterm infants (9). Reducing pain may have corollary effects on the number of critical events experienced by an infant in the NICU (38). Research has shown that pain is attenuated when the mother's heartbeat and voice are present during the procedure (39).

Neurodevelopmental outcomes are also positively influenced. Electroencephalogram (EEG) studies indicate that neonates exposed to their mother's voice exhibit stronger neural responses in the auditory cortex, suggesting improved language acquisition potential (37, 40) Moreover, neonates receiving maternal voice stimulation showed higher oxytocin levels, indicative of reduced stress and protective mechanism against early pain perception (41). Early auditory exposure may enhance synaptic plasticity in brain regions associated with language and emotional regulation, laying the foundation for improved cognitive and emotional resilience as the infant matures. While improvements in EEG activity and auditory cortex maturation have been described, these findings are not universal. Some studies have reported modest or inconsistent neural responses, underscoring the need for further studies in inferring long-term developmental benefit (19).

Improvement in preterm infants’ feeding process, feeding performance and physical growth rates have also been reported with maternal sound stimulation (42). Infants exposed to maternal voice stimuli demonstrate greater rooting and sucking reflexes, leading to higher breastfeeding initiation rates. This primary effect of breastfeeding promotion may lead to secondary effects for the baby. This lends itself to increases in intelligence, stability of body mass index (BMI), reductions in asthma, and protection against mortality.

From a maternal perspective, these interventions have been shown to mitigate the stress and anxiety experienced by mothers of NICU infants by enabling acoustic connectivity between mother and child (43). Reduced anxiety can also enhance breastmilk production, benefiting both mother and infant. Strengthening maternal confidence through a personalized care approach can improve post-discharge caregiving. Similarly, enabling the father to share his voice and heartbeat may help overcome cultural and practical NICU visitation barriers, fostering a stronger paternal bond (44). The intervention may also restore routine for siblings by allowing mothers to spend more time at home and facilitate early sibling-infant bonding through auditory connections (45). Playing recorded sounds from parents, siblings and wider family for the infant in NICU can also serve as a nurturing bridge between the infant and its family. By reducing separation stress for all family members, sound stimulation has the potential to enhance overall family well-being during the NICU journey.

From a clinical perspective, these interventions align with existing developmental care models such as the Newborn Individualized Developmental Care and Assessment Program (NIDCAP) (31). By integrating maternal sound stimulation into standard NICU protocols, hospitals can enhance individualized care plans that prioritize sensory regulation and parental engagement. Furthermore, the low-cost and non-invasive nature of these interventions makes it a feasible intervention for both high-resource and resource-limited settings, addressing disparities in neonatal care globally.

### 5.3 Practical considerations for clinical implementation

Whenever feasible, live parental vocal interaction, particularly during kangaroo care should be considered the preferred and developmentally optimal mode of delivery, with recordings serving as a complementary strategy when continuous parental presence is not possible. Practical implementation of recorded maternal sound stimulation requires attention to recording quality, safe sound levels (<45 dB), and individualized timing based on the infant's behavioural cues. Clinical teams can support parents in creating short, calming recordings (e.g., singing, talking, heartbeat capture) and ensure monitoring of exposure and infant responses in the short term as well as longer term outcomes for the infant and the family. Integration into developmental care plans should involve, physiotherapists, occupational therapists, nurses, speech therapists and neonatologists to optimize safety and efficacy.

### 5.4 Limitations of the existing evidence

Despite promising findings, the current evidence base for maternal sound stimulation has several limitations. Many studies are small, single-centre, or lack adequate control groups, which increases susceptibility to selection and performance bias. Interventions vary widely—some have use live voice, others have used recorded speech, singing, heartbeat sounds or white noise; making cross-study comparison difficult. Blinding is inherently challenging in behavioural interventions, which may influence outcome reporting. Several studies measure short-term physiological outcomes but provide limited insight into longer-term neurodevelopmental, emotional, or language outcomes. Additionally, contradictory evidence exists: some trials report minimal or no improvement in feeding or physiological stability, suggesting that benefits may depend on gestational age, NICU context, or acoustic quality of the intervention. These methodological constraints highlight the need for larger, multi-centre RCTs with standardized protocols and long-term follow-up as well as specific focus on both the optimum quality as well as quantity of sound stimulation.

## 6 Conclusion

By replicating the fetal auditory environment through a suite of tools and measures, we may be able to mitigate sensory deprivation, reduce stress, and promote neurodevelopment for infants looked after in NICUs. The relative ease, low cost and non-invasive nature of this intervention along with widespread clinical applicability, with potential benefits extending to infant health and parental well-being, suggest that the neonatal community needs to give serious consideration to its implementation and on-going study. Despite promising results, the overall quality of evidence remains variable, and reported benefits should be interpreted within the context of small samples, heterogeneous study designs, and inconsistent outcomes across trials. Future research should focus on longitudinal studies tracking language acquisition, executive function, and mental and developmental health outcomes and technological advancements to optimize its efficacy. The integration of controlled auditory stimulation in neonatal care settings holds great promise for addressing the sensory deficits imposed by premature birth, ultimately fostering healthier developmental trajectories and more positive long-term outcomes.
